# Network analysis for count data with excess zeros

**DOI:** 10.1186/s12863-017-0561-z

**Published:** 2017-11-06

**Authors:** Hosik Choi, Jungsoo Gim, Sungho Won, You Jin Kim, Sunghoon Kwon, Changyi Park

**Affiliations:** 1 Department of Applied Statistics, Kyonggi University, Suwon, 16227 Korea; 2 Institute of Health and Environment, Seoul National University, Seoul, 08826 Korea; 3 Graduate School of Public Health, Seoul National University, 08826Seoul, Korea; 4 Department of Nutritional Science and Food Management, Ewha Womans University, Seoul, 03760 Korea; 5 Department of Applied Statistics, Konkuk University, Seoul, 05029 Korea; 6 Department of Statistics, University of Seoul, Seoul, 02504 Korea

**Keywords:** Count data, EM algorithm, Network, Zero inflation

## Abstract

**Background:**

Undirected graphical models or Markov random fields have been a popular class of models for representing conditional dependence relationships between nodes. In particular, Markov networks help us to understand complex interactions between genes in biological processes of a cell. Local Poisson models seem to be promising in modeling positive as well as negative dependencies for count data. Furthermore, when zero counts are more frequent than are expected, excess zeros should be considered in the model.

**Methods:**

We present a penalized Poisson graphical model for zero inflated count data and derive an expectation-maximization (EM) algorithm built on coordinate descent. Our method is shown to be effective through simulated and real data analysis.

**Results:**

Results from the simulated data indicate that our method outperforms the local Poisson graphical model in the presence of excess zeros. In an application to a RNA sequencing data, we also investigate the gender effect by comparing the estimated networks according to different genders. Our method may help us in identifying biological pathways linked to sex hormone regulation and thus understanding underlying mechanisms of the gender differences.

**Conclusions:**

We have presented a penalized version of zero inflated spatial Poisson regression and derive an efficient EM algorithm built on coordinate descent. We discuss possible improvements of our method as well as potential research directions associated with our findings from the RNA sequencing data.

## Background

Graphical models help us to explore relationships between nodes in graphs. Undirected graphical models or Markov random fields have been a popular class of models for representing conditional dependence relationships between nodes. Examples include Gaussian graphical models for continuous data, Ising model for binary data, and multinomial graphical models. These Markov networks help us to understand complex interactions between genes in biological processes of a cell and have been well studied in bioinformatics. Examples of Markov networks in learning the network structure from microarray and next generation sequencing data include [1–4]. For more details on Markov network inference, see those and the references therein.

The main focus of this study is to infer the network structure for a count data. The auto-Poisson model in [5] is a natural extension of univariate Poisson distribution. However it can model only negative dependencies, so that the conditional distributions define a unique joint distribution consistently. Yang et al. [6] propose variants of the auto-Poisson model such as truncated, quadratic, and sublinear Poisson graphical models(PGM). However none of them provide a satisfactory answer to the question of how to specify a consistent joint graphical model for count data capturing both positive and negative dependencies. Allen and Liu [4] consider a local PGM (LPGM). The LPGM does not have a consistent joint graphical model, but it has the local Markov property and thus the zero coefficient of an edge weight between two nodes implies the conditional independence of the two nodes given the others. žitnik and Zupan [7] consider a latent factor Poisson model and [8] propose to learn conditional dependence structures for binary and Poisson data via marginal loss functions. Also a semiparametric Guassian copula, called the nonparanormal graphical model (NPGM), has been proposed [9].

In practice, zero counts are sometimes more frequent than are expected under a univariate Poisson distribution. In such cases, a zero-inflated Poisson (ZIP) distribution is often adopted. Applications of ZIP models include modeling of defects in quality control [10] and alcoholism and substance abuse in medicine [11]. Extensions of a ZIP model in different frameworks are well-studied in the literature. Dobbie and Welsh [12] extend the two component approach in [13] for serially correlated count data exhibiting extra zeros. Monod [14] develops a zero-inflated spatial Poisson (ZISP) model. Buu et al. [11] study variable selection methods such as LASSO and one-step SCAD for ZIP regression models. For computation, a local linear approximation (LLA) is adopted. The LLA algorithm fails to converge particularly with small sample sizes because it requires fitting unpenalized ZIP regression models. Wang et al. [15] propose an expectation maximization (EM) algorithm [16] for a penalized ZIP regression model built on coordinate descent algorithms. The EM algorithm seems to have some advantages over the LLA algorithm in numerical convergence and tuning.

In this paper, we are interested in the construction of graphical models for count data, particularly, with excessive zeros. To this end, we propose a penalized version the ZISP model in [14] called zero inflated local Poisson graphical model (ZILPGM) and derive an EM algorithm built on coordinate descent as in [15]. We show the effectiveness of our method on simulated and real data. In an application to a RNA sequencing data, we investigate the gender effect by comparing the estimated networks according to different genders. It has been well noted that gender is one of the major contributors in the differentiation of gene expression profiles [17, 18] and various sexually dimorphic phenotypes, most of which result from hormonal differences [19]. It was reported that transcriptome study could be predicted to represent a different promising approach for the identification of biological pathways linked to sex hormone regulation and the analysis of associated gene regulatory networks [20]. However, the elucidation of underlying mechanisms of the gender differences is still an area of interest and intense investigation.

The paper is organized as follows. In “Methods” section, we propose a new graph learning method based on ZISP and provide an efficient EM type numerical algorithm. In “Results” section, we compare performances of our method with LPGM on simulated and real data sets. Some discussions and concluding remarks are given in “Conclusions” section.

## Methods

In this section, we present our graph learning method based on a penalization of the ZISP in [14] and derive an efficient EM algorithm for its computation.

### Zero inflated local Poisson graphical model

Let *N* denote the number of observations and *p* denote the number of variables or nodes. Denote $\mathcal {G}=(V, E)$, where *V*={1,…,*p*} is the set of vertices or nodes and *E* is the set of edges. We use uppercase letters such as *X* and *Z* when we refer to random variables. Observations are written in lowercase. For example, *x*_*i*_ denote *i*th observation of *X*. Vectors and matrices are represented by boldface and blackboard boldface letters, respectively. Define $\mathbb {X}=(x_{ij})_{N \times p}$, where *x*_*ij*_ is generated from two latent components with zero and Poisson states. Let *z*_*ij*_ be a latent variable such that *z*_*ij*_=1 if *x*_*ij*_ is from zero state and *z*_*ij*_=0 if *x*_*ij*_ is from Poisson state. *z*_*ij*_ follows a Bernoulli distribution with *π*_*j*_. Let *I*(·) denotes an indicator function. Then the ZISP model in [14] is defined by

1 $$ {}\mathbb{P}\left(X_{j}=x_{j} | X_{k} \,=\,x_{k}, k\neq j\right)\!=\pi_{j}I(x_{j}=0) + \left(1-\pi_{j}\right) \frac{e^{-\mu_{j}} \mu_{j}^{x_{j}}}{x_{j}!},  $$

where $\mu _{j} = \exp \left (\beta _{j} + \sum _{k\neq j} \beta _{jk}x_{k} \right)$, *β*_*j*_ is an intercept adjusting for *X*_*j*_, and *β*_*jk*_ is the parameter accounting for the conditional relation between *X*_*j*_ and *X*_*k*_.

Due to the zero inflation term in the conditional probability, the situation becomes more complicated in our case than in LPGM. Because the important part is the pairwise interaction term in the pairwise-only dependency models, the situation is basically similar. In order to have a valid joint distribution, the coefficient for the interaction term *β*_*jk*_ should be non-positive. As in the LPGM, we do not solve the issue of negative parameters in the Poisson graphical model. Note that any existing approaches (e.g. in [6]) do not succeed in giving a satisfactory answer to the consistency issue. Rather, we focus not on the consistency issue but on the practical issue of estimating positive as well as negative dependencies as in LPGM.

In order to learn graph structures, we consider the minimization of the penalized pseudo log-likelihood of (1) in the general weighted LASSO form:

2 $$\begin{array}{*{20}l} - \frac{1}{N} \sum_{i=1}^{N} \sum_{j=1}^{p}\log& \left(\pi_{j}I(x_{ij}=0) + \left(1-\pi_{j}\right) \frac{e^{-\mu_{ij}} \mu_{ij}^{x_{ij}}}{x_{ij}!} \right) \\ & +\lambda \sum_{j=1}^{p} \sum_{k\neq j} w_{jk} |\beta_{jk}|, \end{array} $$

where $\mu _{ij} = \exp \left (\beta _{j} + \sum _{k\neq j} \beta _{jk}x_{ik} \right)$, *λ*≥0 is the penalty parameter, and *w*_*jk*_≥0 is an appropriate weight. As in [4], we can select the tuning parameter using the stability selection criterion in [21]. More specifically, we select the optimal *λ* is selected from 30 equal-spaced grid points in log scale on [ *λ* ^max^,*λ* ^min^], where $\lambda ^{\text {max}}=\max _{j \in \{1,\cdots,p\}}\max _{k\neq j} \frac {1}{N} \sum _{i=1}^{N} x_{ik}x_{ij}$ and *λ* ^min^=*λ* ^max^×10^−4^. For each *j*, we fit poisson regression using glmnet. Then *w*_*jk*_=1 for covariates with nonzero coefficients. Otherwise, *w*_*jk*_ is set to be sufficiently large value, e.g., 10^5^. Note that the purpose of the penalization is to select spatial neighbors. If *β*_*jk*_=0, then *X*_*j*_ and *X*_*k*_ is declared to be conditionally independent of the other variables.

The penalized pseudo log-likelihood in (2) is separable with respect to the coordinate index. Hence minimizing (2) is equivalent to separately minimizing the *p* coordinate functions:

3 $$\begin{array}{*{20}l} -\frac{1}{N}\sum_{i=1}^{N} \log& \left(\pi_{j}I(x_{ij}=0) + \left(1-\pi_{j}\right) \frac{e^{-\mu_{ij}} \mu_{ij}^{x_{ij}}}{x_{ij}!} \right)\\ &+\lambda \sum_{k\neq j} w_{jk}|\beta_{jk}|\, j=1,\ldots,p. \end{array} $$

Details on the algorithm is discussed later in this section. Once we solve (3), we can estimate the graph structure from the estimated set of edges: $\hat {E} = \{ (j,k) : \hat {\beta }_{jk} \neq 0~\text {or}~\hat {\beta }_{kj} \neq 0, j \neq k\}$. We devise an EM algorithm as in [15] to minimize (3).

### Computational algorithm

Let $\mathcal {O}_{j}=\{i:x_{ij} = 0\}$ and $\mathcal {P}_{j}=\{i:x_{ij} \neq 0\}.$ The negative log-likelihood function in (2) is the sum of

$$\begin{array}{*{20}l} {}l_{j} =-&\sum_{i=1}^{N} \log\left(\pi_{j}I(x_{ij}=0)+ \left(1-\pi_{j}\right) \frac{e^{-\mu_{ij}} \mu_{ij}^{x_{ij}}}{x_{ij}!} \right) \\ =-&\sum_{i \in \mathcal{O}_{j}} \log\left({\vphantom{\sum_{i=1}^{N}}}\pi_{j}+ \left(1-\pi_{j}\right){e^{-\mu_{ij}}} \right) \\-&\sum_{i \in \mathcal{P}_{j}} \log\left(\left(1-\pi_{j}\right) \frac{e^{-\mu_{ij}} \mu_{ij}^{x_{ij}}}{x_{ij}!} \right)\\ =-&\sum_{i \in \mathcal{O}_{j}} \log\left(\frac{\pi_{j}}{1-\pi_{j}}+{e^{-\mu_{ij}}} \right) -\sum_{i=1}^{N} \log\left(1-\pi_{j}\right)\\ +&\sum_{i \in \mathcal{P}_{j}} \left(\mu_{ij}-x_{ij} \log\mu_{ij}\right) +\sum_{i \in \mathcal{P}_{j}}\log x_{ij}! \end{array} $$

for *j*=1,…,*p*. However, it is difficult to maximize this likelihood directly because the score function of $-\sum _{i \in \mathcal {O}_{j}} \log \Big ({\pi _{j}}/\left (1-\pi _{j}\right) + {e^{-\mu _{ij}}} \Big)$ cannot be simplified [14, 22].

Instead of a direct optimization of the likelihood function, we express the likelihood function as a mixture distribution by introducing a latent variable and derive an EM algorithm.

Define ***β***_−*j*_=(*β*_0_,(*β*_*k*_)_*k*≠*j*_))^*T*^ and *x*_*i*,−*j*_=(1,(*x*_*ik*_)_*k*≠*j*_))^*T*^. The log-likelihood function with respect to complete data can be written as

$$\begin{array}{*{20}l} l^{c}_{j} =&-\sum_{i=1}^{N}z_{ij}\log \pi_{j}-\sum_{i=1}^{N}\left(1-z_{ij}\right)\\& \times\left(x_{ij}\mathbf{x}_{i,-j}^{T}\boldsymbol{\beta}_{-j}-\exp\left(\mathbf{x}_{i,-j}^{T}\boldsymbol{\beta}_{-j}\right)-\log x_{ij}!\right)\\ &\equiv l_{j}^{c1}+l_{j}^{c2}. \end{array} $$

The decomposed likelihood function in the above can be easily maximized via an EM algorithm alternating between the expectation of the complete data likelihood over the latent variable *z*_*ij*_ and the maximization of the likelihood given *z*_*ij*_’s.

Define the responsibility of zero state for *j*th variable on *i*th observation at *m*th step as $z_{ij}^{(m)}=\mathbb {E} \left (z_{ij}|x_{ij},\boldsymbol {\beta }_{-j}^{(m)}\right)$ and the probability of zero state at *m*th step as

$$ \pi_{j}^{(m)} = \frac{1}{n} \sum_{i=1}^{n} \left(I\left(x_{ij}=0\right)-I\left(x_{ij}=0\right) \left(1-z_{ij}^{(m)}\right) \right). $$

Our EM algorithm alternates the following steps until convergence.

- E-step: Estimate *z*_*ij*_ by its conditional mean $z_{ij}^{(m)}$ given data and parameters from the previous step.
  $$z_{ij}^{(m)}= \left\{ \begin{array}{ll} \frac{\pi_{j}^{(m)}}{\pi_{j}^{(m)}+\left(1-\pi_{j}^{(m)}\right)\exp\left(-\mu_{ij}^{(m)}\right)} & \text{if } x_{ij}=0,\\ 0, & \text{if } x_{ij}=1,2,\ldots \end{array}\right. $$
- M-step : Estimate $\boldsymbol {\beta }_{-j}^{(m)}$.

Here we set the initial values for our EM iteration as $\pi _{j}^{(0)}=$ the number of zeros of *j*th variable /*n* for *j*=1,⋯,*p* and $\boldsymbol {\beta }_{-j}^{(0)}=\mathbf {0}$.

Now let us discuss the estimation of $\boldsymbol {\beta }_{-j}^{(m)}$ in detail. For each variable, we use the Majorize-Minimization (MM) algorithm in [23], which extends the central idea of EM algorithms to situations not necessarily involving missing data nor even maximum likelihood estimation. A function *g*(*θ*|*θ*_*m*_) is said to majorize a function *f*(*θ*) at *θ*_*m*_ provided that *f*(*θ*_*m*_)=*g*(*θ*_*m*_|*θ*_*m*_) and *f*(*θ*)≤*g*(*θ*|*θ*_*m*_) for *θ*≠*θ*_*m*_. The key idea is that the surrogate majorizing function *g*(*θ*|*θ*_*m*_) is minimized iteratively, instead of the original objective function *f*(*θ*) with the nonquadratic log likelihood and the nondifferentiable sparsity inducing penalty [23]. The MM algorithm starts from an initial guess, *θ*_0_. Let *θ*_*m*+1_ denote the minimizer of the surrogate *g*(*θ*|*θ*_*m*_). Then the following inequalities hold:

$$\begin{array}{@{}rcl@{}} f(\theta_{m+1}) \le g(\theta_{m+1}|\theta_{m}) \le g(\theta_{m}|\theta_{m}) =f(\theta_{m}). \end{array} $$

The above inequality can easily be shown by definition of *θ*_*m*+1_ and the majorization conditions. The descent property makes the MM algorithm numerically stable [24].

The objective to maximize is $l_{j}^{c2} = -\sum _{i=1}^{N}\left (1-z_{ij}\right) \left (x_{ij} \boldsymbol {x}_{i,-j}^{T}\boldsymbol {\beta }_{-j}-\exp \left (\boldsymbol {x}_{i,-j}^{T}\boldsymbol {\beta }_{-j}\right)-\log x_{ij}!\right)$ whose first and second derivatives with respect to ***β***_−*j*_ are

$$\begin{array}{@{}rcl@{}} \frac{\partial l_{c}}{\partial \boldsymbol{\beta}_{-j}} &=& -\sum_{i=1}^{N} \left(1-z_{ij}\right)\left(x_{ij}-\mu_{ij}\right) \boldsymbol{x}_{i,-j},\\ \frac{\partial l_{c}}{\partial \boldsymbol{\beta}_{-j}\partial \boldsymbol{\beta}_{-j}^{T}} &=&\sum_{i=1}^{N} (1-z_{ij})\mu_{ij} \boldsymbol{x}_{i,-j}\boldsymbol{x}_{i,-j}^{T}. \end{array} $$

Let *X*_−*j*_=(***x***_1,−*j*_,…,***x***_*N*,−*j*_)^*T*^. Define $\boldsymbol {b}^{(m)}=\left (\left (1-z_{1j}\right)\left (x_{1j}-\mu _{1j}^{(m)}\right), \ldots, \left (1-z_{Nj}\right)\left (x_{Nj}-\mu _{Nj}^{(m)}\right)\right)$ and $E^{(m)}=X_{-j}^{T}\text {diag}\left (\left (1-z_{1j}\right)\mu _{1j}^{(m)}, \ldots, \left (1-z_{Nj}\right)\mu _{Nj}^{(m)}\right)X_{-j}$. If we ignore additive constants, the quadratic approximation of the objective function at ${\hat{\boldsymbol{\beta}}}_{-j}^{(m)}$ yields

$$\begin{array}{*{20}l} l_{j}^{c2} &\approx \frac{1}{2}\left(\boldsymbol{\beta}_{-j}-\hat{\boldsymbol{\beta}}_{-j}^{(m)}\right)^{T} E^{(m)}\left(\boldsymbol{\beta}_{-j}-\hat{\boldsymbol{\beta}}_{-j}^{(m)}\right)\\&-\left(\boldsymbol{b}^{(m)}\right)^{T}X_{-j} \left(\boldsymbol{\beta}_{-j}-\hat{\boldsymbol{\beta}}_{-j}^{(m)}\right)\\ &\le \frac{\sigma^{(m)}}{2}\left(\boldsymbol{\beta}_{-j}-\hat{\boldsymbol{\beta}}_{-j}^{(m)}\right)^{T}X_{-j}^{T}X_{-j} \left(\boldsymbol{\beta}_{-j}-\hat{\boldsymbol{\beta}}_{-j}^{(m)}\right)\\&-\left(\boldsymbol{b}^{(m)}\right)^{T}{X}_{-j} \left(\boldsymbol{\beta}_{-j}-\hat{\boldsymbol{\beta}}_{-j}^{(m)}\right) \end{array} $$

for an appropriate *σ* ^(*m*)^. To find an appropriate upper bound, we may set *σ* ^(*m*)^ as the maximum of $\left (1-z_{ij}^{(m)}\right)\mu _{ij}^{(m)}$ for *i*=1,…,*N*. We can easily show that

$$\sigma^{(m)}{X_{-j}^{T}X_{-j}}- E^{(m)} $$

is a positive definite matrix. The upper bound can be expressed as

$$l_{j}^{c2} \le \frac{\sigma^{(m)}}{2} \|\boldsymbol{w}_{-j}^{(m)}-X_{-j}\beta_{-j}\|_{2}^{2}, $$

where $\boldsymbol {w}_{-j}^{(m)}=X_{-j}\hat {\boldsymbol {\beta }}_{-j}^{(m)}+{\sigma ^{(m)}}^{-1}{b}^{(m)}$. The majorized problem is written as

4 $$\begin{array}{@{}rcl@{}}  \min_{\scriptsize \boldsymbol{\beta}_{-j} \in \mathbb{R}^{p}} \left(\frac{1}{2} \left\|\boldsymbol{w}_{-j}^{(m)}-X_{-j}\boldsymbol{\beta}_{-j}\right\|_{2}^{2}+\frac{\lambda}{\sigma^{(m)}}\sum_{k \neq j} w_{jk} |\beta_{k}|\right). \end{array} $$

Up to a constant depending not on ***β***_−*j*_ but on ${\hat{\boldsymbol{\beta}}}_{-j}^{(m)}$, the function in the minimization problem (4) majorizes $l_{j}^{c2}$. Hence we achieve the property, guaranteeing the convergence of the algorithm for $\boldsymbol {\beta }_{-j}^{(m)}$ in M-step.

## Results

In this section, we illustrate that our method is effective through a simulation study by comparing the performances of our method, LPGM, and NPGM on simulated data. Then we apply our method to a RNA sequencing data. Also we investigate the gender effect by comparing the estimated networks according to different genders.

### Simulation

To simulate data from a Poisson network with excess zeros, we modify the data generation scheme in [4] slightly. Let *X*∈{0,1,⋯,*∞*}^*N*×*p*^ denote *n* independent observations from a Poisson network with *p* nodes. The data generation model is given as

$$ X=YB+E, $$

where *Y* is a *N*×(*p*+*pC*_2_) matrix with *Y*_*ij*_∼*iid*Poisson(*λ*_true_) and *E* is a *N*×*p* matrix with *E*_*ij*_∼*iid*Poisson(*λ*_noise_). The coefficient matrix *B* encoding the true underlying graph structure denoted by the adjacency matrix *A*∈{0,1}^*p*×*p*^ is defined as

$$B=\left[I_{p};P \odot \left(1_{p} \text{tri}(A)^{T}\right)\right]^{T}, $$

where *P* is the *p*×*pC*_2_ pairwise permutation matrix, ⊙ denotes the element-wise product, and tri(*A*) is the *pC*_2_×1 vectorized upper triangular portion of the adjacency matrix. Each of off-diagonal elements in *A* is randomly generated from Bernoulli(*ρ*), where *ρ* is the sparsity parameter for the network defined as the number of active edges in *A* divided by the number of all possible edges between the nodes. In order to make *X*_*ij*_’s to have excess zeros, we multiply each of *X*_*ij*_ by a random variate from Bernoulli(*π*) for *i*=1,…,*N* and *j*=1,…,*p*. As an abuse of notation, we denote the final matrix containing zero inflated Poisson counts as *X*.

The Poisson rates were set as *λ*_true_=1.5 and *λ*_noise_=0.5. And we have experimented at different levels of *N*(=50,100,150), *p*(=10,20,30), *π*(=0*%*,10*%*,20*%*), and *ρ*(=.2,.3,.4). At each experimental condition, we generated data according to the above scheme and compared the areas under the curve (AUC) from ZILPGM, LPGM, and NPGM. AUC can be obtained in this way. If we regard active and in-active edges in *A* as positive and negative examples in a binary classification, then we can compute true positive rate (TPR) as the fraction of edges found by a method that are in the true underlying network structure *A*. False positive rate (FPR) can obtained analogously. Receiver operating characteristic (ROC) curve and AUC can be obtained from TPR and FPR. To assess the variabilities, we replicated the process of generating data and computing AUC’s 100 times. In Tables 1 and 2, average AUC’s of ZILPGM and LPGM with their standard errors in parentheses and *p*-value from the paired sign rank test on AUC’s over 100 replications are reported.

**Table 1:** Average AUCs for ZILPGM, LPGM, and NPGM on simulated data with their standard errors in parentheses

*π*			0%			10%			20%		
*p*	*N*	*ρ*	ZILPGM	LPGM	NPGM	ZILPGM	LPGM	NPGM	ZILPGM	LPGM	NPGM
		.2	.9945	.9944	.9826	.8657	.8454	.8040	.7852	.7476	.6871
			(.0009)	(.0009)	(.0026)	(.0079)	(.0090)	(.0095)	(.0092)	(.0100)	(.0104)
	50	.3	.8972	.8974	.8880	.7619	.7244	.6894	.6820	.6374	.5862
			(.0053)	(.0053)	(.0061)	(.0073)	(.0087)	(.0092)	(.0085)	(.0090)	(.0094)
		.4	.7748	.7749	.7534	.6948	.6526	.5919	.6428	.6105	.5342
			(.0075)	(.0076)	(.0083)	(.0089)	(.0089)	(.0092)	(.0077)	(.0079)	(.0083)
		.2	.9948	.9948	.9949	.9491	.9379	.9316	.8744	.8487	.8222
			(.0013)	(.0013)	(.0012)	(.0050)	(.0056)	(.0058)	(.0080)	(.0087)	(.0093)
10	100	.3	.9342	.9341	.9284	.8337	.7759	.7341	.7575	.6864	.6283
			(.0043)	(.0043)	(.0049)	(.0055)	(.0067)	(.0075)	(.0070)	(.0084)	(.0088)
		.4	.8188	.8188	.8182	.7255	.6522	.6207	.6589	.5992	.5600
			(.0064)	(.0065)	(.0067)	(.0070)	(.0086)	(.0090)	(.0093)	(.0085)	(.0089)
		.2	.9974	.9974	.9919	.9765	.9586	.9088	.9331	.8893	.7897
			(.0004)	(.0004)	(.0011)	(.0024)	(.0037)	(.0059)	(.0043)	(.0061)	(.0086)
	150	.3	.9762	.9762	.9618	.9546	.9103	.8330	.9008	.8361	.7196
			(.0027)	(.0027)	(.0036)	(.0034)	(.0052)	(.0068)	(.0047)	(.0064)	(.0083)
		.4	.9217	.9216	.9158	.8454	.7646	.6939	.7846	.7046	.6129
			(.0039)	(.0039)	(.0044)	(.0057)	(.0069)	(.0077)	(.0061)	(.0080)	(.0088)
		.2	.8183	.8182	.7778	.7146	.6847	.6098	.6701	.6368	.5432
			(.0042)	(.0042)	(.0045)	(.0048)	(.0052)	(.0055)	(.0043)	(.0053)	(.0054)
	50	.3	.7088	.7091	.6608	.6602	.6318	.5426	.6374	.6188	.5190
			(.0041)	(.0041)	(.0047)	(.0044)	(.0045)	(.0047)	(.0039)	(.0043)	(.0046)
		.4	.6237	.6239	.5902	.6071	.5881	.5206	.5883	.5811	.5071
			(.0040)	(.0040)	(.0045)	(.0040)	(.0038)	(.0037)	(.0039)	(.0043)	(.0045)
		.2	.9530	.9527	.9191	.8511	.8048	.7091	.7824	.7297	.6052
			(.0019)	(.0019)	(.0026)	(.0037)	(.0046)	(.0056)	(.0043)	(.0052)	(.0063)
20	100	.3	.8043	.8043	.7666	.7050	.6555	.5738	.6575	.6241	.5270
			(.0034)	(.0034)	(.0038)	(.0038)	(.0039)	(.0042)	(.0041)	(.0041)	(.0046)
		.4	.7146	.7147	.6982	.6298	.5876	.5406	.5932	.5651	.5093
			(.0039)	(.0039)	(.0042)	(.0039)	(.0041)	(.0042)	(.0039)	(.0041)	(.0043)
		.2	.9440	.9440	.9239	.8163	.7430	.6929	.7387	.6634	.5996
			(.0019)	(.0019)	(.0024)	(.0038)	(.0047)	(.0049)	(.0042)	(.0049)	(.0055)
	150	.3	.8230	.8229	.8200	.6820	.6019	.5821	.6224	.5603	.5360
			(.0032)	(.0032)	(.0035)	(.0042)	(.0043)	(.0045)	(.0039)	(.0042)	(.0041)
		.4	.7237	.7239	.7215	.6256	.5634	.5411	.5939	.5443	.5155
			(.0039)	(.0039)	(.0039)	(.0039)	(.0039)	(.0041)	(.0043)	(.0038)	(.0039)
		.2	.6931	.6932	.6494	.6389	.6198	.5385	.6124	.6067	.5123
			(.0031)	(.0031)	(.0033)	(.0032)	(.0031)	(.0033)	(.0028)	(.0028)	(.0031)
	50	.3	.5875	.5874	.5716	.5580	.5443	.5069	.5494	.5436	.5014
			(.0029)	(.0029)	(.0031)	(.0025)	(.0027)	(.0031)	(.0025)	(.0027)	(.0028)
		.4	.5623	.5624	.5420	.5578	.5467	.5013	.5537	.5517	.5009
			(.0028)	(.0028)	(.0029)	(.0025)	(.0027)	(.0030)	(.0026)	(.0028)	(.0030)
		.2	.8050	.8051	.7651	.6949	.6447	.5675	.6506	.6214	.5295
			(.0029)	(.0029)	(.0030)	(.0029)	(.0032)	(.0036)	(.0031)	(.0032)	(.0033)
30	100	.3	.7015	.7016	.6675	.6289	.5910	.5191	.6025	.5900	.5096
			(.0028)	(.0028)	(.0030)	(.0025)	(.0030)	(.0031)	(.0027)	(.0031)	(.0032)
		.4	.6180	.6183	.5975	.5758	.5564	.5071	.5649	.5551	.5007
			(.0029)	(.0029)	(.0030)	(.0026)	(.0026)	(.0027)	(.0025)	(.0028)	(.0029)
		.2	.8316	.8315	.8151	.6811	.6130	.5775	.6306	.5688	.5246
			(.0026)	(.0026)	(.0028)	(.0031)	(.0033)	(.0035)	(.0032)	(.0032)	(.0033)
	150	.3	.7112	.7114	.6965	.6151	.5672	.5269	.5919	.5526	.5056
			(.0029)	(.0028)	(.0030)	(.0027)	(.0029)	(.0031)	(.0024)	(.0027)	(.0027)
		.4	.6287	.6288	.6211	.5735	.5359	.5058	.5557	.5329	.5002
			(.0026)	(.0026)	(.0027)	(.0028)	(.0027)	(.0028)	(.0026)	(.0026)	(.0026)

Sparsity means the network sparsity, i.e., the number of edges divided by the number of all possible pairs of nodes

**Table 2:** Comparison of ZILPGM, LPGM, and NPGM on simulated data

*π*			0%			10%			20%		
			ZILPGM	ZILPGM	LPGM	ZILPGM	ZILPGM	LPGM	ZILPGM	ZILPGM	LPGM
*p*	*N*	*ρ*	vs.	vs.	vs.	vs.	vs.	vs.	vs.	vs.	vs.
			LPGM	NPGM	NPGM	LPGM	NPGM	NPGM	LPGM	NPGM	NPGM
		.2	0.518	0.000	0.000	0.055	0.000	0.001	0.004	0.000	0.000
	50	.3	0.509	0.156	0.152	0.001	0.000	0.004	0.000	0.000	0.000
		.4	0.507	0.043	0.042	0.000	0.000	0.000	0.002	0.000	0.000
		.2	0.497	0.481	0.483	0.079	0.011	0.186	0.011	0.000	0.012
10	100	.3	0.499	0.306	0.308	0.000	0.000	0.000	0.000	0.000	0.000
		.4	0.503	0.473	0.462	0.000	0.000	0.004	0.000	0.000	0.001
		.2	0.518	0.000	0.000	0.000	0.000	0.000	0.000	0.000	0.000
	150	.3	0.493	0.000	0.000	0.000	0.000	0.000	0.000	0.000	0.000
		.4	0.488	0.208	0.214	0.000	0.000	0.000	0.000	0.000	0.000
		.2	0.480	0.000	0.000	0.000	0.000	0.000	0.000	0.000	0.000
	50	.3	0.517	0.000	0.000	0.000	0.000	0.000	0.001	0.000	0.000
		.4	0.511	0.000	0.000	0.001	0.000	0.000	0.129	0.000	0.000
		.2	0.445	0.000	0.000	0.000	0.000	0.000	0.000	0.000	0.000
20	100	.3	0.484	0.000	0.000	0.000	0.000	0.000	0.000	0.000	0.000
		.4	0.492	0.004	0.004	0.000	0.000	0.000	0.000	0.000	0.000
		.2	0.491	0.000	0.000	0.000	0.000	0.000	0.000	0.000	0.000
	150	.3	0.458	0.364	0.371	0.000	0.000	0.001	0.000	0.000	0.000
		.4	0.505	0.401	0.383	0.000	0.000	0.000	0.000	0.000	0.000
		.2	0.488	0.000	0.000	0.000	0.000	0.000	0.060	0.000	0.000
	50	.3	0.488	0.000	0.000	0.000	0.000	0.000	0.099	0.000	0.000
		.4	0.514	0.000	0.000	0.002	0.000	0.000	0.325	0.000	0.000
		.2	0.490	0.000	0.000	0.000	0.000	0.000	0.000	0.000	0.000
30	100	.3	0.508	0.000	0.000	0.000	0.000	0.000	0.000	0.000	0.000
		.4	0.538	0.000	0.000	0.000	0.000	0.000	0.005	0.000	0.000
		.2	0.474	0.000	0.000	0.000	0.000	0.000	0.000	0.000	0.000
	150	.3	0.517	0.000	0.000	0.000	0.000	0.000	0.000	0.000	0.000
		.4	0.513	0.016	0.015	0.000	0.000	0.000	0.000	0.000	0.000

The *p*-value has been obtained from the sign rank test on AUC’s from ZILPGM, LPGM, and NPGM over 100 replications

Let us consider the effects of each factor with the other factors held fixed. As *ρ* increased (or the network became dense), AUC’s of all the compared methods have decreased. Similarly, as the dimension *p* grew larger, their AUCs became smaller. As the sample size *N* grows, AUC’s tends to improve. However the tendency is sometimes not so clear. Now consider the effect of excess zeros. When there is no zero inflation (*π*=0), AUC’s from ZILPGM, LPGM, and NPGM were not significantly different. When we have zero inflations (*π*=0.1,0.2), ZILPGM seems to significantly outperform LPGM and NPGM. NPGM seems to be outperformed by LPGM. The gaps between AUC’s from ZILPGM and LPGM when *π*=0.2 was not necessarily larger than that when *π*=0.1. A potential explanation for this phenomenon follows. As *π* increases, we have more zero counts in the data and thus the estimation accuracy for the mixing parameter will improve. Meanwhile, the estimation accuracy for the Poisson parameters can degrade because Poisson parameters are learned from nonzero counts. The tradeoff between these two estimation errors may occur at a certain level of *π*.

### Chromosome data

To investigate the validity of the proposed method, we applied it to the RNA sequencing data in the form of a count matrix that contains the number of mapped reads for 60 normal individuals in [25]. We selected 899 genes in the sex chromosomes, i.e., X and Y, first. Each of 899 genes has many zero counts. For a gene with almost all the counts equal to zero, its mixing parameter is estimated as one. To reduce the computation, we have reduced the original data to a data of dimension *n*=60,*p*=360 by keeping genes with the number of non-zero counts less than or equal to one.

**Fig. 1:**
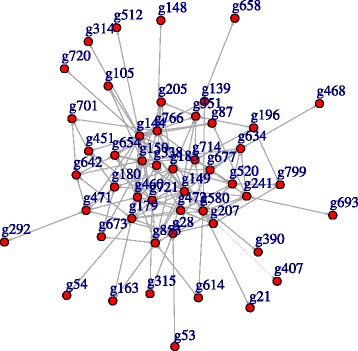
Estimated chromosome network

Figure 1 shows the estimate for the network structure from our method. While 49 genes are clustered together, the other genes remain isolated. Top ranked genes are shown in Table 3 according to their degrees. Note that the degree of a gene is the number of edges being incident upon the gene. We further identified the function of genes with large degree. By GO-BP annotation, NDUFA1 and NDUFB11 are involved in mitochondrial electron transport chain (especially complex I), which affects the capacity for the production of ATP through oxidative phosphorylation. GO annotations related to MID1IP1 and PIM2 are protein C-terminus binding and transferase activity, respectively. Proteins with these functions should highly interact with other proteins to control regulation process in cells. Meanwhile, genes with small degrees, SYAP1 and P2RY10, involved in PI3K/Akt signaling and G-protein coupled receptor (GPCR) activity, respectively [26]. GPCR activate the PI3K/Akt signaling pathway involved in the cellular responses including metabolism, proliferation, apoptosis, and survival [27].

**Table 3:** Top ranked genes with their degrees for chromosome data

ID	Gene	Degree	ID	Gene	Degree	ID	Gene	Degree
g144	MID1IP1	44	g634	PBDC1	14	g163	OTUD5	8
g149	NDUFA1	31	g714	STS	14	g148	NSDHL	7
g580	MSN	31	g180	RAB33A	12	g292	APEX2	7
g150	NDUFB11	30	g471	HSD17B10	12	g512	MAGT1	7
g721	SYN1	29	g520	MMGT1	12	g673	RNF113A	7
g766	UBQLN2	29	g799	ZBTB33	12	g390	COX7B	6
g460	GPC4	28	g314	BEX3	11	g614	PNPLA4	6
g179	PIM2	22	g451	GLUD2	11	g693	SLC10A3	6
g677	SEPT6	22	g472	HPRT1	11	g87	GPKOW	5
g853	RPS4Y1	22	g642	PGRMC1	11	g407	EBP	5
g196	ARHGEF6	20	g654	PLP2	11	g468	HCCS	5
g241	TSR2	20	g701	SLC9A6	11	g658	P2RY10	5
g28	CXCR3	17	g205	SASH3	10	g139	MAGEH1	4
g207	SH3BGRL	17	g53	ELK1	9	g21	BCAP31	3
g351	XCorf21	17	g105	LAGE3	9	g720	SYAP1	3
g185	RAB9A	16	g315	BEX4	9			
g338	CHST7	14	g54	ERCC6L	8			

**Fig. 2:**
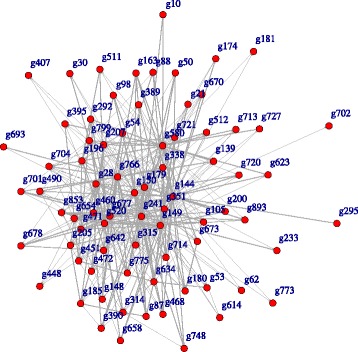
Estimated chromosome network for male group

**Fig. 3:**
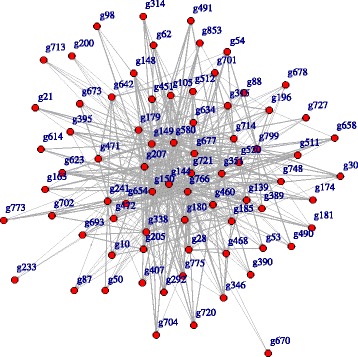
Estimated chromosome network for female group

Now let us investigate the effect of gender. In order to compare the networks for different gender groups with 27 males and 33 females, we applied our method to each of gender groups separately. The estimated networks for male and female groups are shown in Figs. 2 and 3. The differentially expressed genes in each group are listed in Table 4. Originally, a differentially expressed gene in a treatment and control groups is a gene with mean expression levels in those groups are significantly different. Although our method is not explicitly related to a hypothesis testing for comparing mean levels, it is implicitly related to a hypothesis testing for conditional independence of counts between genes through a regularized graph learning method on count data. So, in this sense, we call a gene differentially expressed in male and female groups if it appears only one of the the estimated networks for male or female groups. For example, ARMCX1 was selected as a node in the network of the male group and CLIC2 was selected in the network of the female group.

**Table 4:** Genes differential expressed in male and female groups

Only male	Only female
g295 (ARMCX1)	g346 (CLIC2)
g448 (GRPR)	g491 (KLHL34)
g893 (TMSB4Y)	

The ARMCX1 gene encodes a member of the ALEX family of proteins and is located on the X chromosome. It was reported that downregulated ARMCX1 transcripts have been found to be significantly reduced prostate cancer and may play a role of tumor suppressor gene [28, 29]. CLIC2, a member of the glutathione S-transferase structural family and a suppressor of cardiac ryanodine receptor (RyR2) Ca2+ channels located in the membrane of the sarcoplasmic reticulum, is controlled by redox-dependent processes and would allow to limit cellular damage in terms of oxidative stress [30]. Above mentioned cellular oxidant detoxification and glutathione metabolic process could inhibit age-related deterioration, protect the human neuronal cells, and regulate the expression of many genes primarily involved during immune system activities and inflammatory responses [31].

Following GO functional enrichment analysis, genes differentially expressed in the male group included SLC9A7, PLP2, MAGT1, COX7B, STK26, CYBB, MMGT1, BCAP31, and SLC9A6, whereas genes differentially expressed in the female group were RAB33A and UBQLN2. The differentially expressed genes in the male group are involved in the ion transport-related pathways, whereas the differentially expressed genes in the female group are involved in the regulation of autophagosome assembly.

It has been implicated that ion transport pathways may play a key role in the male reproductive potential, such as capacitation and the acrosome reaction, which are critical steps in sperm physiology preparing for fertilization [32]. On the other hand, it has been investigated on the formation of an autophagosome stimulated by oxidative or metabolic stress taking into account the sex/gender disparities in terms of immunity and inflammation [33–35]. Furthermore, these advantages of women in immunity and inflammation have been well known and these phenotypic differences in immune responses from males result from direct genetic differences [34, 36].

## Discussion

In this paper, we propose a penalized version of zero inflated spatial Poisson regression and derive an efficient EM algorithm built on coordinate descent. On simulated data, our method was shown to yield competitive performances in terms of AUC. Particularly, in the presence of excess zeros, our method outperformed LPGM, which is a state of art method in learning graph structures for count data. Note that one may apply the likelihood ratio test for non-nested hypotheses in [37] in order to test for excess zeros on each node. Also we have applied our method to the chromosome data to infer its network structure. Constructing the networks for different genders, we identified the genes differentially expressed in the male and female groups.

There are several issues we have not addressed in this paper. First, one may study the properties our estimators. For Guassian graphical models, asymptotic properties of the estimators are rather well studied in the literature. For example, [38] study asymptotic normality and optimalities in the estimation of Gaussian graphical models. Monod [14] provides sufficient conditions for the consistency of the MLE for ZISP model and discusses some properties such as asymptotic normality and efficiency of the MLE. Because our model is based on a penalization of ZISP model, the results in [14] will provide a starting point for studying properties of the estimators. Particularly, in our case, the properties of the estimators for the incidence matrix rather than the coefficients are of interest. Second, our method can be applied to construct biological networks as well as other networks for count data with excess zeros. Examples include user-ratings, spatial incidence of a disease or crime, word-document counts, and others. Third, one may also extend our model to Poisson graphical models with multiple-inflations as in [39]. Still another direction is to generalize our model to other distributions such as negative binomial and gamma distributions.

## Conclusions

In the present study, expression of ARMCX1 and CLIC2 turned out to be different according to gender. Very little is known about the functional properties of these two genes, this could make ARMCX1 and CLIC2 the possible candidates of medical relevance, such as prostate cancer in male [28, 29] and oxidative stress-related diseases for female [40]. Therefore, further evidences seem to be necessary for identifying gene expression patterns and validating its diagnostic potential that differentiated patients with relevant diseases from healthy controls in each sex in the population-based cohorts and, afterwards, it will be translated to clinical practice with its diagnostic impact.

## Abbreviations

AUC: Area under the curve
EM: Expectation-maximization
FPR: false positive rate
GPCR: G-protein coupled receptor
LLA: Local linear approximation
LPGM: Local Poisson graphical model
MM: Majorization minimization
NPGM: Nonparanormal graphical model
PGM: Poisson graphical model
ROC: Receiver operating characteristic
TPR: True positive rate
ZILPGM: Zero-inflated local Poisson graphical model
ZIP: Zero-inflated Poisson
ZISP: Zero-inflated spatial Poisson
